# Robust, Flame-Retardant, and Anti-Corrosive Waterborne Polyurethane Enabled by a PN Synergistic Flame-Retardant Containing Benzimidazole and Phosphinate Groups

**DOI:** 10.3390/polym15102400

**Published:** 2023-05-22

**Authors:** Li-Ping Zhang, Zhen-Guo Zhao, Yuan-Yuan Huang, Chang-Jian Zhu, Xing Cao, Yan-Peng Ni

**Affiliations:** Institute of Functional Textiles and Advanced Materials, Qingdao Key Laboratory of Flame-Retardant Textile Materials, National Engineering Research Center for Advanced Fire-Safety Materials D & A (Shandong), State Key Laboratory of Bio-Fibers and Eco-textiles, College of Textiles & Clothing, Qingdao University, Qingdao 266071, China

**Keywords:** waterborne polyurethanes, flame reaction, corrosion resistance, mechanical properties, emulsion stability

## Abstract

Waterborne polyurethanes (WPUs) have attracted great interest owing to their environmentally friendly properties, and are wildly applied in production and daily life. However, waterborne polyurethanes are flammable. Up to now, the challenge remains to prepare WPUs with excellent flame resistance, high emulsion stability, and outstanding mechanical properties. Herein, a novel flame-retardant additive, 2-hydroxyethan-1-aminium (2-(1H-benzo[d]imidazol-2-yl)ethyl)(phenyl)phosphinate (BIEP-ETA) has been synthesized and applied to improve the flame resistance of WPUs, which has both phosphorus nitrogen synergistic effect and the ability to form hydrogen bonds with WPUs. The WPU blends (WPU/FRs) exhibited a positive fire-retardant effect in both the vapor and condensed phases, with significantly improved self-extinguishing performance and reduced heat release value. Interestingly, thanks to the good compatibility between BIEP-ETA and WPUs, WPU/FRs not only have higher emulsion stability, but also have better mechanical properties with synchronously improved tensile strength and toughness. Moreover, WPU/FRs also exhibit excellent potential as a corrosion-resistant coating.

## 1. Introduction

Waterborne polyurethanes (WPUs) exhibit many advantages over solvent-based polyurethanes, such as high security and reliability, low VOCs (low volatile organic compounds), less damage to the coated surface, easy operation, and transportation, and have been widely applied as an alternative solution for solvent-based polyurethanes in the fields of coatings, leather finishing agents, fabric finishing agents, adhesives, paper, and fiber sizing agent, etc. [1,2,3]. Nevertheless, waterborne polyurethanes have the same high flammability characteristics as most organic polymer materials, which greatly limits the further expansion of their applications [4,5]. Accordingly, the research on the flame-retardancy of WPUs have received widespread attention from academia and industry.

Over the years, a great deal of effort has been made on improving the flame resistance of WPUs. In general, the way to achieve flame resistance of WPUs is to add fire retardants through physical blending or introduce flame-retardant groups through chemical reactions [6,7]. For reactive flame-retardant modification of WPUs, the main difficulties are that how to make the introduction of flame-retardant have no obvious influence on the preparation process of dispersions, including polymerization and emulsification, nor destroy the emulsion stability and mechanical properties [8]. Currently, many attempts and efforts have been put into practice, yet still suffered from many problems. Although chemical modifications can endow WPUs with flame-retardancy, the mechanical properties (strength, toughness, hardness, etc.) or emulsion stability are generally deteriorated, making it difficult to meet the requirements of practical applications [9]. In brief, reactive flame-retardant modifications of WPUs make it difficult to achieve a balance between flame-retardancy and comprehensive performance.

Due to its advantages of not involving chemical reactions, simple process, convenient operation, relatively wide range of sources, and low cost, additive flame-retardant modification is generally the preferred choice in the industry and dominates in practical applications [10,11]. The challenge faced by additive modification is the compatibility between fire retardants and waterborne polyurethanes, and poor compatibility may lead to demulsification of the dispersions and damage to mechanical properties [12,13]. Although some researchers have proposed various fire-retardant systems to alleviate the problem, such as nanoparticle systems, ionic liquids, etc. [14], it is difficult to avoid the poor efficiency on flame-retardancy or the negative effects of comprehensive performance. For example, Ma et al. added modified LDH nanoparticles as a flame-retardant for WPUs and obtained samples with a limiting oxygen index (LOI) value of only 25.9% when the LDH usage was as high as 10%. Moreover, poor dispersion and compatibility also bring about a weakening in the strength and toughness of WPUs [15]. Zhao et al. applied an ionic liquid flame-retardant “[Dmim]Tos” containing phosphorus and nitrogen element to waterborne polyurethanes. Although excellent flame-retardancy was achieved at lower usage levels, unfortunately, the mechanical strength also decreased, attributed to the plasticizing effect of the “[Dmim]Tos” [16]. Obviously, modifiers with high flame-retardancy efficiency, good compatibility and even improved comprehensive performance of WPUs are the focus of research on additive flame-retardants.

For halogen-free flame-retardancy, phosphorus is considered one of the most effective elements for WPUs. Moreover, the combination of phosphorus and nitrogen-containing flame-retardants often result in better synergistic flame-retardancy [17,18]. Therefore, for waterborne polyurethanes, developing phosphorus/nitrogen (P/N) synergistic flame-retardants is a more ideal choice. From the perspective of improving mechanical properties, according to literature reports, adding fillers with hydrogen bonding sites can enhance intermolecular forces and obtain WPUs with high mechanical properties when constructing polyurethane systems [19,20]. Moreover, based on the hydrogen bonding complexation strategy, polymer materials with good dispersibility, good compatibility, and excellent mechanical properties can be prepared through flame-retardants containing hydrogen bond donors and acceptors [21].

Therefore, in this work, we synthesized a new P/N synergistic flame-retardant, 2-hydroxyethan-1-aminium (2-(1H-benzo[d]imidazol-2-yl)ethyl)(phenyl)phosphinate (BIEP-ETA), by combining structures rich in hydrogen bond donors and acceptors such as benzimidazole groups and amine groups with phosphorus-containing groups. Then, it was physically mixed into the WPU dispersions to prepare a range of waterborne polyurethanes WPU/FRs. The emulsion stability of WPU/FRs dispersions, thermal and mechanical properties, flame-retardancy, burning behaviors, transparency, and flame-retardant mechanism were investigated. As expected, benefiting from the good compatibility between BIEP-ETA and WPUs, as well as the P–N synergistic effects on flame-retardancy between phosphinate groups and the benzimidazole groups, WPU/FRs exhibit satisfactory storage stability, excellent flame-retardancy, and mechanical properties. Moreover, WPU/FRs also exhibit excellent potential as a corrosion-resistant coating.

## 2. Materials and Methods

### 2.1. Materials

Isophorone diisocyanate (IPDI) and (2-carboxyethyl)phenylphosphinic acid (CEPPA) were supported by Aladdin Bio-Chem Technology Co., Ltd. (Shanghai, China). Polypropylene glycol (PPG, Mw = 2000 g mol^−1^), triethylamine (TEA), 1,4-Butanediol (BDO), and ethanolamine were obtained from Zhengye Reagent Instrument Co. Ltd. (Qingdao, China). 2,2-Bis(hydroxymethyl)propionic acid (DMPA), o-phenylenediamine (OPD) and dibutyltin dilaurate (DBTDL) were brought from Macklin Biochemical Co., Ltd. (Shanghai, China).

PPG was dried at 100 °C in a vacuum for 3 h in advance to remove moisture inside. IPDI and BDO were dehydrated with 4A molecular sieves for a week before use.

### 2.2. Preparation of WPU Dispersions

In the preparation of WPUs, PPG as an oligomer polyol mainly provided soft segment function in the polymer structure and could react with IPDI-containing -NCO groups to prepare the prepolymer. Firstly, 46.68 g PPG2000 and 140 g IPDI were fed into a four-neck flask equipped with mechanical stirring and stirred for 10 min in a nitrogen atmosphere to obtain a homogeneous mixture. Then, DBTDL that acted as the catalyst was added dropwise and the prepolymerization reaction continued for 1 h at 80 °C, and the prepolymer was prepared. Subsequently, 9.86 g DMPA (the hydrophilic chain extender) and 0.59 g BDO (the small molecule chain extender) were added to the system and the chain extension reaction continued for another 3 h, which could increase the molecular weight of polyurethanes. In addition, TEA, as a neutralizing base, can undergo a neutralization reaction with DMPA to ionize it, which allows the polyurethane to be dispersed in water. The polyurethanes were neutralized with 7.44 g TEA at 50 °C for 1 h. In the preparation process, acetone was used as an auxiliary solvent to decrease the system viscosity. Next, 600 mL deionized water was added to the system slowly and polyurethanes were emulsified under high-speed stirring (1300 rpm) for 1 h. Finally, the WPU dispersions were prepared through removing acetone in vacuum.

### 2.3. Synthesis of 2-Hydroxyethan-1-aminium (2-(1H-benzo[d]imidazol-2-yl)ethyl)(phenyl)phosphinate (BIEP-ETA)

The synthesis route of BIEP-ETA was presented in Scheme S1. 64.25 g (2-carboxyethyl)phenylphosphinic acid (CEPPA) and 32.40 g O-phenylenediamine (OPD) were dissolved in 150 mL hydrochloric acid solution (4 mol L^−1^) and then the mixture refluxed at 80 °C for 6 h. After that, the mixture was neutralized to a pH value of 7.0 at room temperature. Through filtration, washing with deionized water, and drying in vacuum, (2-(1H-benzo[d]imidazol-2-yl)ethyl)(phenyl)phosphinic acid (BIEPA) was obtained. Further, BIEPA and ethanolamine were reacted at 80 °C for 2 h according to a molar ratio of 1:1 with a solvent of ethanol. Finally, the product (BIEP-ETA) was dried by rotating evaporation to remove the ethanol and subsequently in a vacuum at 80 °C for 10 h.

**Figure 1:**
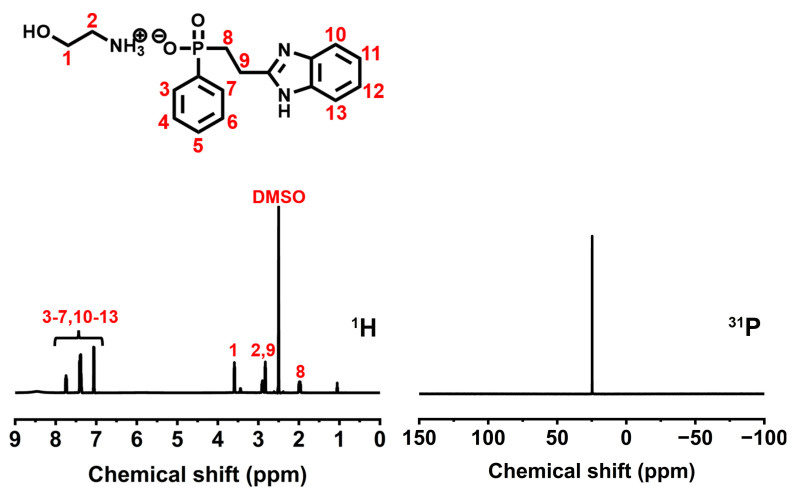
^1^H (**left**) and ^31^P (**right**) spectra of BIEP-ETA.

**Figure 2:**
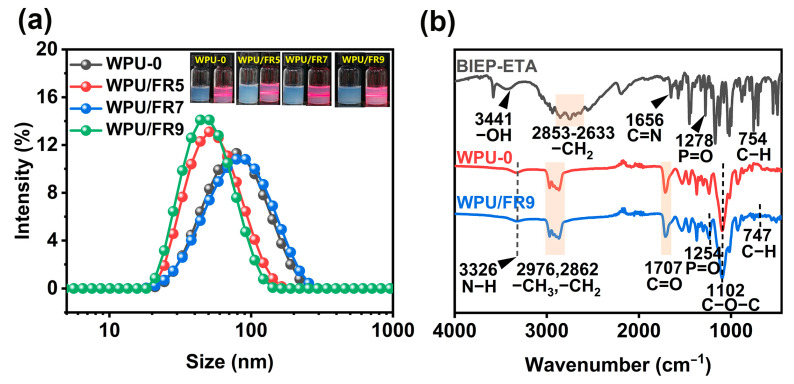
(**a**) Particle size distribution curves and appearance of WPU dispersions and (**b**) infrared spectra of BIEP-ETA, WPU, and WPU/FR9.

BIEP-ETA: white power, yield: 77%. The NMR and FTIR spectra of BIEP-ETA are displayed in Figure 1 and Figure 2, respectively. FTIR: 3441 cm^−1^ (−OH), 2633–2853 cm^−1^ (−CH_2_−), 1656 cm^−1^ (−C=N−), 1278 cm^−1^ (−P=O), 754 cm^−1^ (−C-H). ^1^H NMR (400 MHz, DMSO, ppm): 7.06–7.74 (Ar−H,9H), 3.59 (−CH_2_-O−,2H), 2.89 (−CH_2_-N−,2H), 2.83 (−CH_2_−C=N,2H), 1.98 (−CH_2_−P=O,2H). ^31^P NMR (161.9 MHz, DMSO, ppm): 24.74. The detailed information of ^1^H NMR and ^31^P NMR are shown in Figure S1.

### 2.4. Preparation of WPU/FRs

WPU/FRs dispersions were fabricated through solution mixing and the specimens were labeled as WPU/FR5, WPU/FR7, and WPU/FR9, where the numbers 5, 7, and 9 denoted the weight percentages of BIEP-ETA to solid WPU of 5%, 7%, and 9%, respectively. Take the preparation process of WPU/FR9 as an example, 18.40 g BIEP-ETA was completely dissolved into the 400 g deionized water, then WPU dispersion (800.0 g, solid content was 25.0 wt%) was mixed with the BIEP-ETA solution at a speed of 500 rpm for 3 h. Therefore, the solid content of the WPU/FR9 mixture was 20.0 wt%.

Then, 120 g prepared dispersions were poured into the PTFE molds, placed in a well-ventilated area for a week, followed by drying at 60 °C for 24 h. Finally, WPU/FRs films with a thickness of 2 mm were prepared.

### 2.5. Characterization

The particle size distribution and potential of the dispersions were carried out by Malvern Nano ZSE (Malvern, Malvern, UK). Before testing, the emulsions were diluted 1000 times. The emulsion stability was tested by centrifuge at 3000 rpm for 15 min referred to the GB/T 6753.3-1986. The storage stability of the emulsion can be determined to be longer than 6 months if there was no precipitation after centrifugation.

The FTIR spectra of films were recorded with a Thermo Scientific Nicolet iS50 FTIR Spectrometer (Thermo Scientific, Waltham, MA, USA).

X-ray photoelectron spectroscopy (XPS) of residual carbon after combustion were recorded by Axis Supra+ (Shimadzu, Kyoto Prefecture, Japan). The results were calibrated with reference to the binding energy position of the C1s peak.

NMR spectroscopy was performed on a Bruker AVANCE III HD 400 MHz (Bruker Biospin, Karlsruhe, Germany) with DMSO as the solvent.

Differential scanning calorimetry (DSC) was performed on a TA DSC2500 instrument (TA, New Castle, DE, USA) in N_2_ atmosphere. First, the temperature rapidly rose to 100 °C and held for 2 min to eliminate the effect of thermal histories, then the temperature dropped to −80 °C with a cooling rate of 10 °C min^−1^, and finally heated to 100 °C with a same heating rate.

Dynamic mechanical analysis (DMA) was executed on a TA DMA Q800 instrument (TA, New Castle, DE, USA) in a stretching mode from −80 °C to 100 °C with a heating rate of 3 °C min^−1^.

The limiting oxygen index (LOI) tests were implemented using a TTech-GBT2406-2 oxygen index instrument (TESTECH, Suzhou, China) according to ASTM D2863-97 standard. The tested samples were cut into the size of 100 mm × 6.5 mm × 2 mm. The LOI value was defined as the minimum oxygen concentration which made specimens just keep burning where the time was not more than 3 min and the burning length was not more than 5 cm in a mixture of oxygen and nitrogen.

The mechanical properties of the specimens were measured on an INSTRON F5967 (INSTRON, Boston, MA, USA) in accordance with GB/T1040.2-2006. WPU films were into dumbbell-shaped specimens a dumbbell-shaped with a dimensional size of 20 mm × 4 mm × 2 mm.

The spectroscopies of thermogravimetry-infrared spectrometer (TG-IR) were analyzed by PerkinElmer STA6000 thermal analyzer combined using a PerkinElmer Frontier spectrometer (PerkinElmer, Waltham, MA, USA). The temperature of the connected tube between the TG analyzer and FT-IR spectrometer was 260 °C.

The combustion behaviors were investigated by an FTT cone calorimeter (Fire Testing Technology, London, UK) in accordance with the standard of ISO 5660–1. The radiation power was set to 35 kW m^−2^ and the fan flow rate was 24 L s^−1^.

Thermal stability was performed on a TA TGA5500 analyzer (TA, New Castle, DE, USA) with a heating rate of 10 °C min^−1^.

The transparency was analyzed by a UV2700 ultraviolet-visible spectrophotometer (Shimadzu, Kyoto Prefecture, Japan).

The corrosion resistances of the WPU films were measured by a CHI760E electrochemical workstation (Chenhua, Shanghai, China) in 3.5 wt% NaCl electrolyte.

## 3. Results and Discussion

### 3.1. Storage Stability

We first confirmed the chemical structure of BIEP-ETA and WPU through the NMR and FTIR spectra (Figure 1 and Figure 2b). The resonance signals occurring at 3441, 1656, and 1278 cm^−1^ in the FTIR spectrum of BIEP-ETA are attributed to the stretching vibrations of Please confirm that the intended meaning was retained OH, −C=N−, and −P=O bonds, respectively [22,23]. From the ^1^H NMR spectrum of BIEP-ETA, the chemical shifts and integral area ratios of all characteristic peaks can also correspond to BIEP-ETA. Moreover, for the ^31^P NMR spectrum, only one single peak appeared at 32.21 ppm, indicating the presence of only one type of phosphorus. All the above results demonstrate that BIEP-ETA was synthesized successfully.

Storage stability is one of the most important properties of WPU dispersions, which directly affects their use and performance. As expected, BIEP-ETA is well soluble in waterborne polyurethane dispersions and its addition did not cause demulsification of WPU dispersions. From Figure 2a, it can be clearly seen that all dispersions are light blue translucent, and the obvious “Tyndall effect” phenomenon can be observed when the laser beam passes through the dispersion, indicating that the WPU/FRs dispersions are stable colloid with particle sizes ranging from 1 nm to 100 nm [24]. Even after centrifugation test, no precipitation was found, which confirms that all the WPU dispersions can be stably stored for more than half a year. Furthermore, the Z-average particle sizes and zeta potentials of all the WPU dispersions were determined, and the detailed results are listed in Table 1 and Figure 2a. With the addition of BIEP-ETA, the particle size values of WPU/FRs dispersions did not increase significantly, but even smaller, the absolute values of zeta potential were all above 35 mV, demonstrating that their dispersibility and storage stability is decent. Combined with the above analysis results, BIEP-ETA is well compatible with the WPU dispersions and WPU/FRs dispersions have good storage stability.

### 3.2. Thermal and Mechanical Properties

**Figure 3:**
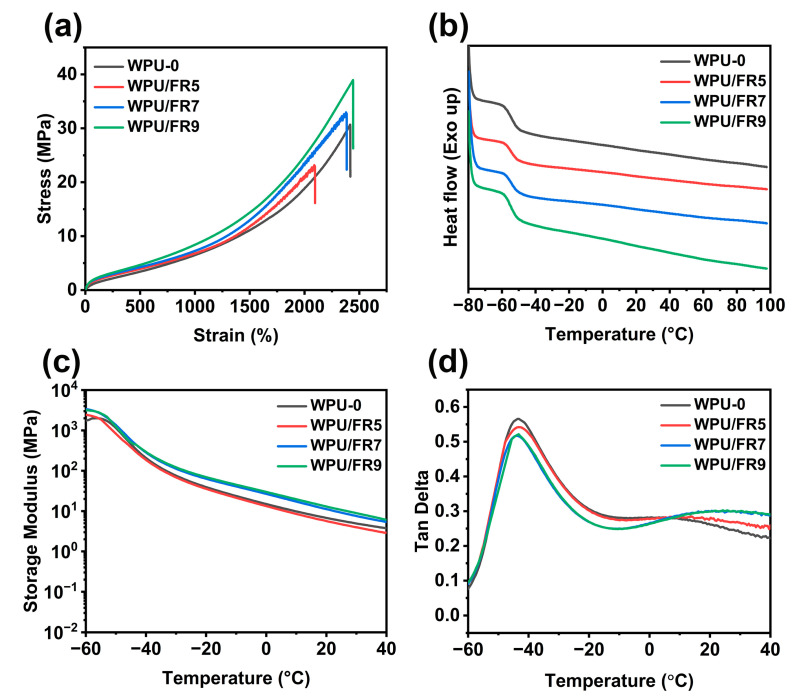
(**a**) The tensile stress–strain curve, (**b**) DSC second heating curves, (**c**) the storage modulus curves, and (**d**) Tan δ curves of WPU-0 and WPU/FRs films.

As we know, microphase separation is the determining factor affecting the mechanical properties of polyurethanes. To understand the effect of BIEP-ETA on the microphase separation, the thermo–physical properties of the specimens were studied by DMA and DSC (shown in Figure 3b–d and Table S1). From Figure 3c, the storage modulus of all the WPU films decreased as the temperature increased. For WPU/FR7 and WPU/FR9, the storage modulus is always higher than that of WPU-0, especially at high temperatures; however, for WPU/FR5, the storage modulus is lower than that of WPU-0 at high temperatures, indicating that a small amount of BIEP-ETA weakens the intermolecular force in the hard segment and more addition will enhance the intermolecular forces in the hard segment. In the DMA test, the temperature value at the maximum signal of tan δ curve is considered as the glass transition temperature (*T*_g_) [25]. All WPU samples have two glass transition temperatures (*T*_g_) values due to the microphase separation between the soft and hard segments [26]. *T*_g_s of the soft segment (*T*_g,s_) were basically maintained at a constant around −43.5 °C, regardless of the BIEP-ETA content, while, *T*_g_s of the hard segment (*T*_g,h_) first decreased and then increased as the BIEP-ETA amount increased. From Figure 3d and Table S1, we can observe peak patterns near 7 °C of WPU-0 and WPU/FR5, *T*_g,h_ of WPU/FR5 slightly lower than that of WPU-0, and *T*_g,h_s of WPU/FR7 and WPU/FR9 move to higher temperature at around 21 °C. Meanwhile, for WPU/FR7 and WPU/FR9, *∆T*_g_s between *T*_g,h_ and *T*_g,s_ have widened, indicating enhancement of the microphase separation.

*T*_g_s of WPU-0 and WPU/FRs are further measured by DSC. It can be observed from Figure 3b that only *T*_g,s_ at low temperature and no other *T*_g,h_ or crystallization peaks are found. *T*_g,_s of all samples are almost identical, which is in agreement with the results of DMA. The *T*_g,_s obtained from DSC show a 10 °C lower than that obtained from DMA. The difference is caused by different testing principles between DSC and DMA, where DSC is based on changes in heat capacity, while DMA is based on changes in thermal mechanical behavior.

To investigate the effect of BIEP-ETA on the mechanical properties, tensile tests are performed. As shown in Figure 3a and Table 2, the tensile strengths of WPU/FRs show a trend of first decreasing and then increasing with the increase of BIEP-ETA addition compared to WPU-0, which are in agreement with the DMA test results. The elongations at break and Young’s modulus also exhibit similar trends. This phenomenon may be ascribed to the characteristics of BIEP-ETA as a polar small molecule and its ability to form multiple hydrogen bonds. Polar small molecules can be inserted between WPU molecular chains, weakening the intermolecular forces between polymer chains, especially the hydrogen bonding between hard segments, playing a role similar to “plasticization” on the one hand [16,27]. On the other hand, BIEP-ETA as a rigid particle has multiple hydrogen bond binding sites (hydroxyl, C=N double bond, N−H bond), which can form multiple hydrogen bonds with waterborne polyurethane molecular chains, enhancing the microphase separation, playing a role in synchronous strengthening and toughening [20,28,29]. When the addition amount is lower, plasticization dominates. As the addition amount increases, the strengthening and toughening are dominant, which is the main reason why there are significant differences both in DMA and tensile tests of WPU/FRs with different contents. For WPU/FR9, the tensile strength and elongation at break are higher than those of WPU-0. Therefore, we can prepare flame-retardant waterborne polyurethanes with higher strength and toughness by controlling the amount of BIEP-ETA added.

### 3.3. Flame-Retardancy and Burning Behaviors

**Figure 4:**
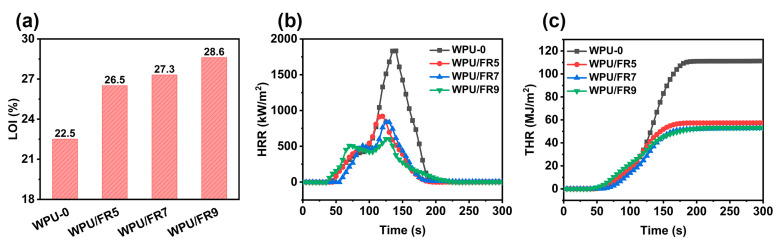
Fire safety performance of WPU-0 and WPU/FRs: (**a**) LOI value, (**b**) HRR curves in cone calorimetry test, and (**c**) THR curves in cone calorimetry test.

Further, the flame resistance of the obtained specimens was evaluated by classic limiting oxygen index (LOI) and cone calorimetry test, and the detailed results are summarized in Figure 4 and Table 3. The limiting oxygen index refers to the minimum concentration of oxygen needed to support the burning of polymers, which is an essential parameter to measure the flammability of materials. The higher the LOI value is, the more difficult the material is to burn and the better the flame-retardancy is [29]. In general, materials exhibiting LOI values greater than 26% will exhibit self-extinguishing performance and are considered as a low flammability material. As shown in Figure 4a, WPU-0 is a flammable material with an LOI value as low as 22.5%. As expected, all WPU/FRs samples showed high LOI values greater than 26%. The LOI value showed a significant increasing trend as more BIEP-ETA was added, in which the values for WPU/FR5, WPU/FR7, and WPU/FR9 were as high as 26.5, 27.3, and 28.6%, respectively.

We conducted cone calorimetry, an oxygen consumption technique providing a burning scenario similar to that of a real fire, to further evaluate the combustion performance of WPU-0 and WPU/FRs (Figure 4b,c and Table 3). The heat release rate (HRR), the peak heat release rate (pHRR), the total heat release (THR), the average effective heat of combustion (Av-EHC), and the maximum average rate of heat emission (MARHE) are considered as the important parameters to evaluate the fire hazards of the materials; the lower the values, the better the fire safety of the materials [20,30,31]. In contrast to WPU-0, the values of these parameters all exhibited a similar significant downward trend, that is, the values decreased with the increase of the BIEP-ETA content of the samples. Specifically, the pHRR and THR values of WPU-0 were 1834 kW/m^2^ and 111 MJ/m^2^; while for WPU/FR9, the two parameters were reduced to 602 kW/m^2^ and 53 MJ/m^2^, which is 67% and 52% lower than that of WPU-0, respectively. Likewise, the values of Av-EHC decreased by 25%, from 33 MJ/kg of WPU-0 to 25 MJ/kg of WPU/FR9, and the values of MARHE also decreased by 51%, from 612 kW/m^2^ of WPU-0 to 301 MJ/kg of WPU/FR9. In addition, after combustion, the WPU-0 sample was almost completely burned with hardly any residue remaining, while WPU/FRs had a significantly residual yield of up to 17% and an obvious and dense carbon layer was formed, which can act as a barrier against the transfer of heat, air, and pyrolysis products and means less fuel generation and feeding-back during combustion. All the above results suggest that WPU/FRs exhibit excellent flame-retardancy, and fire hazards such as heat release have been greatly suppressed.

### 3.4. Flame-Retardant Mechanism Analysis of WPU/FRs

**Figure 5:**
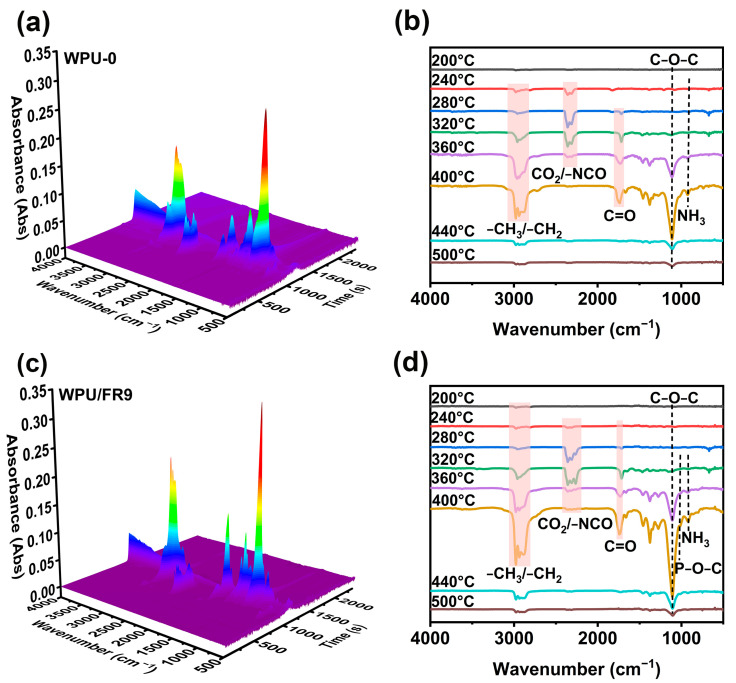
Gas product analysis for WPU-0 and WPU/FRs: (**a**,**c**) Three-dimensional TG-IR spectra and (**b**,**d**) FTIR spectra of degradation products at different temperatures.

To better understand how BIEP-ETA affects flame-retardancy, first, TG-FTIR tests were carried out to analyze the gaseous products in the thermal decomposition process. Figure 5 displays the 3D spectrogram of the gas phase products (a, c) and the chemical structure changes of WPU-0 and WPU/FR9 at different temperatures (b, d) during thermal degradation. As shown in Figure 5, no obvious differences were observed in the gas phase products between WPU-0 and WPU/FR9, implying that the introduction of BIEP-ETA did not alter the degradation path of WPUs. The typical characteristic signals of the decomposition products of waterborne polyurethanes, including 2977–2862 cm^−1^ (hydrocarbon compounds), 2372–2358cm^−1^ (gases containing CO_2_), 2295–2264 cm^−1^, 668 cm^−1^ (NCO groups and HCN), 1738 cm^−1^ (C=O), 1108 cm^−1^ (ethers), and 921 cm^−1^ (NH_3_) [32,33,34], all appear in the spectra of both WPU-0 and WPU/FR9. For WPU-0, gases containing CO_2_ and hydrocarbon compounds are released at 240 °C; this may be the degradation of DMPA. When the temperature reached 280 °C, the characteristic signals of −NCO, −C=O and HCN appeared, the signals of CO_2_ and hydrocarbons increased, suggesting that the hard segment degraded. At 320 °C, the absorption peak of C−O−C gradually appeared, indicating that soft segment of waterborne polyurethanes began to decompose. Peak signals increase to their maximum intensity at 400 °C and then decrease. There were no obvious absorption peaks at 500 °C, indicating that the degradation process of waterborne polyurethanes is basically completed. Most notably, the absorption peak at 1019 cm^−1^ attributed to the stretching band of P−O−C bond was only detected in the spectra of WPU/FR9 rather than in that of WPU-0, indicating that phosphorus-containing compounds with free radical capture activity can be generated during decomposition of WPU/FR9, and also providing evidence for the high vapor-phase flame-retardant activity of BIEP-ETA.

**Figure 6:**
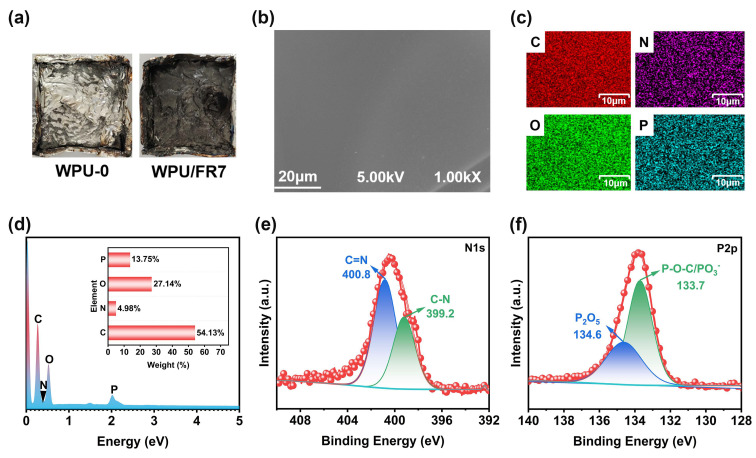
Char layer analysis of WPU/FRs after cone calorimeter test: (**a**) Digital photographs of the burning residues, (**b**) SEM images of the inside residual surface, (**c**) EDX elemental mapping images, and (**d**) element content results obtained from EDX. (**e**,**f**) XPS N_1s_ and P_2p_ spectra of residual char.

Moreover, SEM, EDX, and XPS tests were conducted on the microstructure and composition of the residuals after the CCT test. Given the fact that the WPU-0 samples have been completely burned without any residue, we can only analyze the residual chars of WPU/FRs separately (digital photos of the burning residues are presented in Figure 6a). From SEM micrographs and EDX images (Figure 6b–d), a dense and continuous char layer was observed, which can protect the substrate from heat, oxygen, and inflammable gas. Figure 6c,d show that four elements (C, N, O, and P) are evenly distributed in the residue char, with N and P element contents of 4.98 wt% and 13.75 wt%, respectively, indicating that BIEP-ETA is involved in solid-phase carbonization during the combustion process. To further investigate the chemical composition of char layers, the residual carbons of WPU/FR7 were analyzed by XPS spectroscopy. The N1s and P2p spectra of the residues are presented in Figure 6e,f. For N1s spectrum, the peaks at 400.8 eV and 399.2 eV are attributed to C=N and C−N, respectively [35]; for P2p spectrum, the 134.6 eV and 133.7 eV are assigned to P_2_O_5_ and P−O−C/PO_3_^−^ [36]. These signals are mainly related to the structures generated by dehydration and carbonization during the combustion process.

Combined with the above analysis, we can infer the following possible flame-retardant mechanisms. First, BIEP-ETA degraded and produced many phosphorus-containing free radicals, which can react with active free radicals and interrupt the chain reaction in the burning process. Meanwhile, phosphorus-containing groups decompose to form phosphoric acid. Phosphoric acid is converted into polyphosphate which further generates polyphosphate at high temperatures, which can promote dehydration and charring. Benzimidazole groups also participated in charring, forming a dense and continuous carbon layer on the substrate, thereby hindering the thermal and oxidative diffusion of the underlying material, playing a P–N synergistic flame-retardant effect. BIEP-ETA has positive effects on the fire resistance of waterborne polyurethanes simultaneously in both gaseous and condensed phases.

### 3.5. Thermal Stability and Transparency

**Figure 7:**
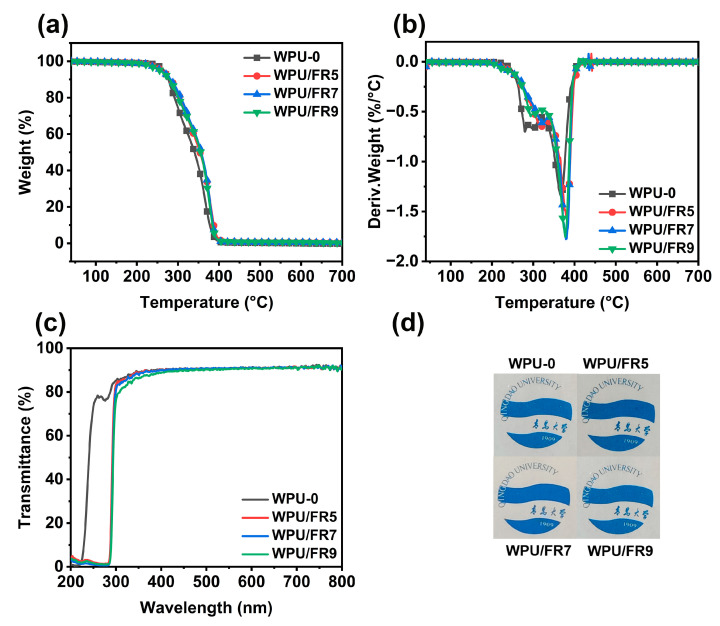
(**a**) TG and (**b**) DTG curves of all the samples in an N_2_ atmosphere, (**c**) UV-vis transmission spectra, and (**d**) digital photos of transparency of all the WPU films.

Figure 7a,b and Table S2 display the TGA results to analyze the thermal stability of WPU-0 and WPU/FRs in an N_2_ atmosphere. As with WPU-0, all the WPU/FRs exhibit a two-step decomposition process. The first and second stages correspond to the degradation of the hard segment and soft segment, respectively. Although the initial decomposition temperatures (*T*_5__%_) of WPU/FRs showed a slight downward, both maximum decomposition temperatures (*T*_max1_ and *T*_max2_) were significantly delayed to higher temperatures, compared with those of WPU-0. This may be due to the early decomposition of BIEP-ETA, which generates abundant phosphorus-based radicals and captures a large number of hydroxyl and hydrogen active radicals during the decomposition process, causing a delay in the decomposition of the polyurethane main chain.

Furthermore, we investigated the influence of BIEP-ETA additive on the transparency of WPU films through the UV-visible absorption spectroscopy. Figure 7c shows the UV-visible transmission curves of all the WPU films. The transmittance of all the WPU samples fluctuated around 90% throughout the entire visible light range. Moreover, from the digital photos in Figure 7d, we can clearly see the patterns and words below the WPU films regardless of how much BIEP-ETA was added or not. In summary, the addition of BIEP-ETA had almost no obvious influence on the transparency and WPU/FRs films have excellent transparency. It is worth noting that the WPU/FRs films exhibited particularly strong absorption in the ultraviolet region below 300 nm, indicating complete resistance to UVc ultraviolet radiation (200–300 nm).

### 3.6. Anticorrosive Property of WPU/FRs

**Figure 8:**
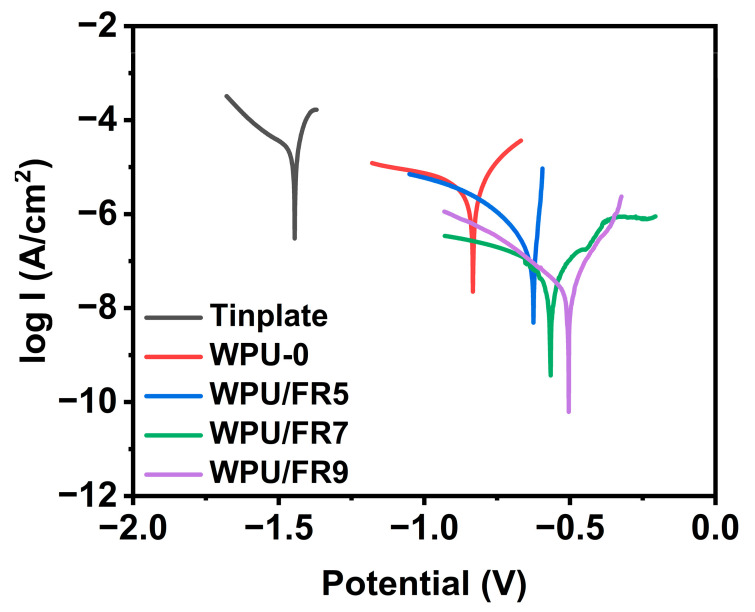
Tafel polarization curves of tinplate substrates coated with WPU-0 and WPU/FRs.

Waterborne polyurethanes can be used as an anti-corrosion coating for metal protection. Coincidentally, as reported, phosphonates and benzimidazole derivatives are also both excellent corrosion inhibitors that can be used for metal corrosion protection [37,38]. Therefore, we have enough reason to believe that WPU/FRs have good potential as an anti-corrosion coating. Subsequently, the effects of BIEP-ETA additive on the anti-corrosion performance of WPU coatings are explored through potentiodynamic polarization studies of tinplate substrates that were uncoated and coated with WPU/FRs in 3.5% NaCl solution. The obtained Tafel polarization curves are shown in Figure 8 and the corresponding corrosion potential (*E*_corr_) and corrosion current (*I*_corr_) parameters are summarized in Table S3. *E*_corr_ and *I*_corr_ can be determined by the intersection of the tangent of the cathodic polarization curve and the tangent of the anodic polarization curve, where the abscissa and ordinate values correspond to *E*_corr_ and *I*_corr_, respectively. As is well-known, *I*_corr_ reflects the speed level of metal corrosion, the smaller the *I*_corr_ value, the slower the metal corrosion; and *E*_corr_ reflects the difficulty of metal corrosion, the higher the *E*_corr_, the less likely the material is to be corroded. From Figure 8 and Table S3, it can be clearly seen that the corrosion protection performance of the WPU/FRs significantly improved with increasing BIEP-ETA content, exhibiting a higher *E*_corr_ and a smaller *I*_corr_ than the sample with WPU-0. It is noteworthy that the *I*_corr_ of the samples coated with WPU/FRs are reduced by two to three orders of magnitude compared to the blank sample and are also one to two orders of magnitude lower than that of the samples coated with WPU-0. This could be attributed to the fact that the phosphinate group and benzimidazole group can form a tight adsorption layer on the metal substrate which helps seal the metal surface and enhance its hydrolysis stability.

## 4. Conclusions

In conclusion, we have successfully synthesized new phosphorus/nitrogen synergistic flame-retardant (BIEP-ETA) and physically blended it with WPU dispersions to prepare various flame-retardant WPU/FRs. Benefiting from the P–N synergistic effects on flame-retardancy between phosphinate groups and the benzimidazole groups, WPU/FRs exhibit excellent flame-retardancy. For WPU/FR9, the LOI value reached as high as 28.6% and its pHRR and THR values decreased by more than half compared to unmodified WPUs. Fortunately, thanks to the good compatibility between BIEP-ETA and WPUs, WPU/FRs also exhibit satisfactory emulsion stability, good transparency, and outstanding mechanical properties. Moreover, WPU/FRs have excellent potential application value as a corrosion-resistant coating. This work not only overcomes the problems of flame-retardancy of WPUs, but also provides a new reference method for solving the compatibility issue between WPUs and functional additives, as well as achieving high-performance of functional waterborne polyurethanes.

## Figures and Tables

**Table 1:** Basic properties of WPU-0 and WPU/FRs dispersions.

Sample	Z-Average Size (nm)	PDI	Zeta Potential (mV)	Centrifugal Stability
WPU-0	74.66	0.257	−37.7	No precipitate
WPU/FR5	53.25	0.193	−36.7	No precipitate
WPU/FR7	77.69	0.226	−41.0	No precipitate
WPU/FR9	46.35	0.111	−39.1	No precipitate

**Table 2:** Data from tensile testing of WPU-0 and WPU/FRs films.

Sample	Tensile Strength (MPa)	Elongation at Break (%)	Young’s Modulus (MPa)
WPU-0	31.5 ± 4.7	2439 ± 107	3.5 ± 0.7
WPU/FR5	24.4 ± 0.7	2138 ± 64	2.2 ± 0.1
WPU/FR7	32.7 ± 1.7	2349 ± 60	2.5 ± 0.3
WPU/FR9	39.1 ± 0.7	2442 ± 13	3.5 ± 0.1

**Table 3:** Data from the cone calorimeter test of WPU-0 and WPU/FRs.

Sample	TTI (s)	pHRR (kW/m^2^)	THR (MJ/m^2^)	Av-EHC (MJ/kg)	MARHE (kW/m^2^)	Char Residue (wt%)
WPU-0	39	1834	111	33	612	0
WPU/FR5	40	920	57	27	345	2.5
WPU/FR7	52	842	52	25	306	15.0
WPU/FR9	33	602	53	25	301	9.0

## Data Availability

The data are available upon request.

## Supplementary Materials

The following supporting information can be downloaded at: https://www.mdpi.com/article/10.3390/polym15102400/s1, Scheme S1: Synthetic route for BIEP-ETA; Figure S1: ^1^H NMR spectra of BIEP-ETA; Figure S2: ^31^P NMR spectra of BIEP-ETA; Table S1: Data from DSC and DMA tests of WPU-0 and WPU/FRs; Table S2: Data from TGA test of WPU-0 and WPU/FRs; Table S3: Tafel polarization data of coated and uncoated tinplate in 3.5% NaCl.
