# Supplementary material for: Robust, Flame-Retardant, and Anti-Corrosive Waterborne Polyurethane Enabled by a PN Synergistic Flame-Retardant Containing Benzimidazole and Phosphinate Groups
Source: Polymers (Basel). 2023 May 22;15(10):2400. doi: 10.3390/polym15102400 (PMC10221596; doi:10.3390/polym15102400)
Supplement: Supplementary file 1 [file polymers-15-02400-s001.zip › polymers-2402987-supplementary.pdf]

Supporting information

Robust, Flame-Retardant and Anti-Corrosive Waterborne Polyurethane Enabled by a P–N Synergistic Flame Retardant Containing Benzimidazole and Phosphinate Groups

Li-Ping Zhang, Zhen-Guo Zhao, Yuan-Yuan Huang, Chang-Jian Zhu, Xing Cao, Yan-Peng Ni*

Institute of Functional Textiles and Advanced Materials, Qingdao Key Laboratory of Flame-Retardant Textile materials, National Engineering Research Center for Advanced Fire-Safety Materials D & A (Shandong), State Key Laboratory of Bio-Fibers and Eco-textiles, College of Textiles & Clothing, Qingdao University, Qingdao 266071, China.

E-mail: polyester-niyanpeng@qdu.edu.cn

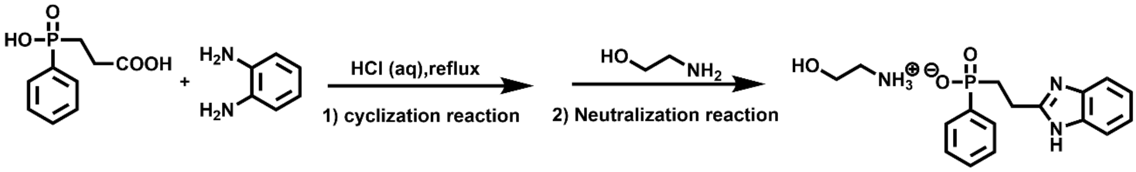

Scheme S1. Synthetic route for BIEP-ETA.

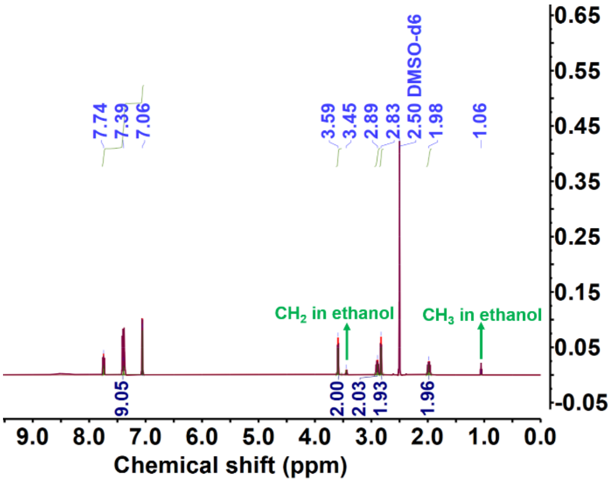

Figure S1. ^{1}H NMR spectra of BIEP-ETA.

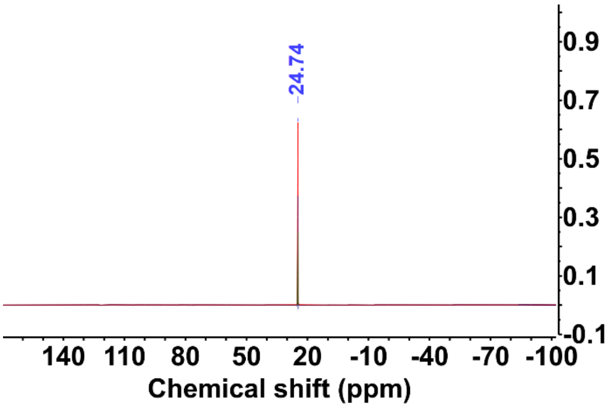

Figure S2. ^{31}P NMR spectra of BIEP-ETA.

Table S1 Data from DSC and DMA tests of WPU-0 and WPU/FRs.

Sample	DSC	DMA		
	$T_{g,s}$ (°C)	$T_{g,s}$ (°C)	$T_{g,h}$ (°C)	Storage Modulus at -60 °C (MPa)
WPU-0	-54.6	-43.4	7.01	1746
WPU/FR5	-54.4	-43.1	6.68	2413
WPU/FR7	-54.1	-43.6	21.50	3367
WPU/FR9	-54.5	-44.2	21.90	3143

Table S2 Data from TGA test of WPU-0 and WPU/FRs.

Sample	$T_{5\%}$ (°C)	T_{max1} (°C)	T_{max2} (°C)	R_{700} (wt%)
WPU-0	266.1	282.7	366.8	0
WPU/FR5	261.8	320.1	381.7	0.31
WPU/FR7	258.6	322.7	379.3	0.32
WPU/FR9	255.2	294.3	377.6	0.25

Table S3 Tafel polarization data of coated and uncoated tinplate in 3.5% NaCl.

Sample	I_{corr} (A/cm^2)	E_{corr} (V)
Tinplate	3.27×10^{-5}	-1.45
WPU-0	4.58×10^{-6}	-0.83
WPU/FR5	1.05×10^{-7}	-0.63
WPU/FR7	7.74×10^{-8}	-0.57
WPU/FR9	3.80×10^{-8}	-0.50
